# Ovarian Function Suppression With Luteinizing Hormone-Releasing Hormone Agonists for the Treatment of Hormone Receptor-Positive Early Breast Cancer in Premenopausal Women

**DOI:** 10.3389/fonc.2021.700722

**Published:** 2021-09-14

**Authors:** Yen-Shen Lu, Andrea Wong, Hee-Jeong Kim

**Affiliations:** ^1^Department of Oncology, National Taiwan University Hospital, Taipei, Taiwan; ^2^Department of Haematology-Oncology, Cancer Science Institute, National University of Singapore, Singapore, Singapore; ^3^Department of Surgery, College of Medicine, Asan Medical Center, Seoul, South Korea

**Keywords:** premenopausal, breast cancer, ovarian function suppression (OFS), ovarian function preservation, endocrine therapy, luteinizing hormone releasing hormone

## Abstract

Chemotherapy and endocrine therapies are mainstays of treatment for early and advanced hormone receptor-positive (HR+) breast cancer. In premenopausal women with HR+ tumors, the benefits of adding ovarian function suppression (OFS) to endocrine therapy have been debated. Consequently, for many years, tamoxifen monotherapy has been the standard of care for endocrine treatment in the adjuvant setting. Recent studies have, however, provided new evidence that, in some premenopausal patients, OFS in combination with tamoxifen or aromatase inhibitors (AIs) can significantly increase survival *versus* tamoxifen alone. Luteinizing hormone-releasing hormone agonists (LHRHa), including goserelin, triptorelin, and leuprorelin, achieve OFS through sustained suppression of the release of follicle-stimulating hormone and luteinizing hormone from the pituitary. In turn, this suppresses production and secretion of estradiol, an ovarian hormone that supports cancer cell growth, survival, and proliferation. In this review, we discuss the clinical evidence supporting the addition of LHRHa to adjuvant endocrine therapies, including tamoxifen and AIs, for premenopausal women with breast cancer. We also discuss the role of LHRHa use in combination with adjuvant chemotherapy to preserve ovarian function and fertility in young patients with breast cancer. Finally, we discuss important practical aspects of the use of LHRHa in breast cancer treatment, including side-effects, patient adherence to treatment, and the use of slow-release, long-acting drug formulations.

## Introduction

Breast cancer is one of the most frequently diagnosed malignancies worldwide. According to the World Health Organization, in 2020 an estimated 2.26 million cases were diagnosed and 685,000 deaths resulted from the disease (1). Although most breast cancer cases occur in postmenopausal women, a substantial proportion occur in premenopausal women under the age of 50 years; estimates range from approximately 20% of all breast cancers in some developed countries, such as the USA, to as many as 50% of all breast cancers in less economically developed countries and some developed countries in Asia (2, 3). This makes breast cancer the most frequently diagnosed malignancy and the leading cause of cancer-related death worldwide in women under 40 years of age (4).

Younger age at diagnosis has long been recognized as a factor associated with higher risk of disease recurrence and death (5, 6), and in premenopausal women, breast cancer is often characterized by tumors with aggressive pathological phenotypes. Evidence from the UK-based Prospective Study of Outcomes in Sporadic and Hereditary breast cancer (POSH) revealed that, at diagnosis, in women aged 18–40 years, median tumor diameter was 22 mm, 58.9% of patients had grade 3 tumors, 50.2% had lymph node-positive disease, and 33.7% had estrogen receptor-negative (ER−) tumors (7). These values are notably higher than those reported in studies of older, postmenopausal women. In line with the more aggressive tumor features, the 5-year overall survival (OS) of patients in the POSH study was worse than that of contemporary patients aged 40–69 years in the UK (81.9% *versus* 89.1–90.4%) (7, 8). Further evidence from retrospective analysis of the Surveillance, Epidemiology and End Results (SEER) database, of over 200,000 patients diagnosed between 1988 and 2003 also showed that women aged under 40 years at diagnosis (n = 15,548) had tumors that were more likely to be larger in size, higher grade, lymph node positive and hormone-receptor-negative (HR−) (9) than those who were older. Thus, the prognosis for young women diagnosed with breast cancer is, in many cases, worse than for older women even though younger patients are often given more intensive treatments (10).

Growing evidence suggests that tumor biology and genetics play a primary role in determining the relatively poorer outcomes in premenopausal women compared with postmenopausal women (6). Numerous studies have identified that tumors in younger patients frequently have different expression patterns of key biomarkers, including HRs, human epidermal growth factor receptor 2 (HER2), and proliferation markers compared with tumors in older, postmenopausal patients (5, 6). In premenopausal women, data from Western countries show that approximately 65–80% of tumors are luminal-type HR-positive (HR+) tumors (5, 7, 11). However, younger patients in these countries have a higher proportion of more aggressive basal-like tumors (also known as triple-negative breast cancer; TNBC) that are ER−, progesterone-receptor-negative (PR−), and HER2-negative (HER2−), as well as a higher proportion of HER2-overexpressing tumors (that are ER−/PR−) than older patients (11–13). A key point to note is that breast cancer in young Asian women has distinctive clinicopathological features that differ from those seen in Western women and therefore it requires different treatment guidelines (14, 15); for example, the probability of being diagnosed with TNBC has been shown to decrease with age in patients from the USA but not in patients from East Asia (15). Nonetheless, HR+ disease remains the most common breast cancer diagnosis in premenopausal women and these patients are, therefore, good candidates for treatment with endocrine therapy in the adjuvant setting.

## Adjuvant Treatment Options for Premenopausal Patients With Breast Cancer

Adjuvant chemotherapy and endocrine therapy are integral to the treatment of early breast cancer and can significantly reduce the risk of death and relapse. In premenopausal breast cancer patients with aggressive TNBC, treatment options are limited, and prognosis is poor relative to patients with HR+ cancers. In these patients, whose tumors are resistant to endocrine therapy and HER2-targeting treatments, cytotoxic chemotherapy remains the only well-validated and approved treatment in the adjuvant setting following surgery (16); although the development of immune checkpoint inhibitors, including programmed cell death 1 (PD-1) and programmed cell death ligand 1 (PD-L1), is changing the treatment landscape for these patients (17).

In HR−/HER2-positive (HER2+) patients, the anti-HER2 agent trastuzumab has transformed disease outcomes and has become the standard of care given as a monotherapy or in combination with other drugs including paclitaxel (16). Newer agents, including pertuzumab (18), trastuzumab emtansine (19), and neratinib (20), have also shown effectiveness in the treatment of patients with HER2+ tumors.

**Figure 1:**
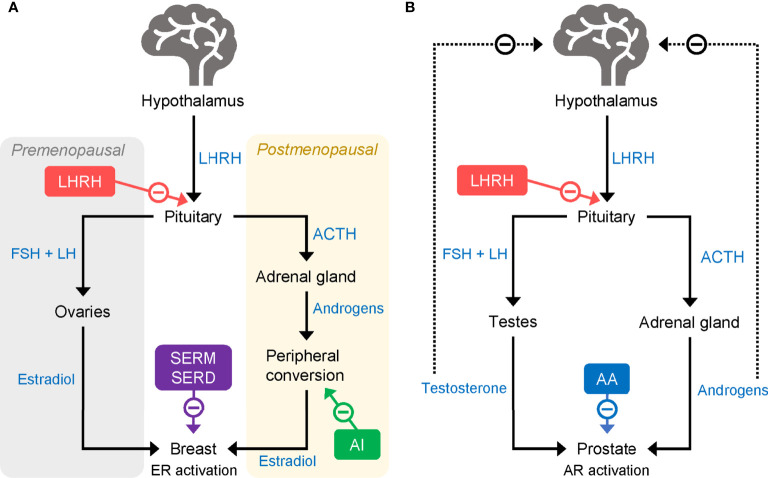
Mode of action of LHRHa in **(A)** breast cancer and **(B)** prostate cancer. AA, abiraterone acetate; ACTH, adrenocorticotropic hormone; AI, aromatase inhibitor; AR, androgen receptor; ER, estrogen receptor; FSH, follicle-stimulating hormone; LH, luteinizing hormone; LHRHa, luteinising hormone-releasing hormone agonist; SERD, selective estrogen receptor degrader; SERM, selective estrogen receptor modulator.

For patients with luminal-type HR+ tumors, adjuvant endocrine therapy is typically the preferred option. Tamoxifen, a selective ER modulator (SERM) (21), has been standard for adjuvant endocrine therapy in both premenopausal and postmenopausal women for many decades (22, 23). By blocking ERs, tamoxifen reduces the mitogenic effects of the ovarian hormone estradiol (E2), helping to prevent cancer cell growth and proliferation (21) (**Figure 1A**). Early trials of tamoxifen, including the Nolvadex Adjuvant Trial Organization (NATO) trial (24, 25), the Cancer Research Campaign Adjuvant Breast (CRCAB) trial (26), and the National Surgical Adjuvant Breast and Bowel Project (NSABP) trial (27, 28), demonstrated clear reductions in risk of disease recurrence and death in patients receiving the drug for between 2 and 5 years in the adjuvant setting. The clinical effectiveness of tamoxifen has been subsequently confirmed by a large meta-analysis (n = 21,457 patients in 20 trials) performed in 2011 by the Early Breast Cancer Trialists’ Collaborative Group (EBCTCG). In 10,645 patients with ER+ disease, 5 years of adjuvant tamoxifen treatment *versus* no adjuvant tamoxifen reduced recurrence rates (RRs) by nearly 50% (RR 0.53) during years 0–4 and 30% (RR 0.68) in years 5–9 of follow up; breast cancer mortality was reduced by approximately 30% during the 15-year follow-up period (22).

Aromatase inhibitors (AIs), including the third-generation compounds letrozole, anastrozole, and exemestane, are a class of endocrine-based therapies commonly used in the adjuvant setting in postmenopausal patients (29). AIs reduce the production of estrogens by suppressing the activity of aromatase enzymes. In premenopausal women, most circulating estrogens are produced in the ovaries, but following menopause aromatases found in fat and muscle tissues are responsible for most estrogen production (**Figure 1A**) (29). Unlike postmenopausal women, premenopausal women have a large amount of ovarian estrogen production under the strong influence of pituitary gonadotropins. AI administration markedly increases gonadotropin release and promotes estrogen-dependent aromatase activity which, in turn, counteracts the effectiveness of AIs in reducing ovarian estrogen production. Thus, in premenopausal women, AIs have limited ability to reduce circulating estrogen and are not typically given without combination with another treatment to suppress ovarian function.

### Ovarian Function Suppression and LHRH Agonists

Ovarian function suppression (OFS) or ablation has been studied in breast cancer for many decades. The relationship between ovarian function and breast cancer was recognized as early as 1882, with the first reported evidence of cancer regression after menopause, and since the 1890’s it has been known that surgical removal of the ovaries in premenopausal patients with breast cancer has the ability to reduce the likelihood of remission (30, 31). Following these pivotal findings, numerous further studies have demonstrated that early menopause, either naturally induced or induced by bilateral oophorectomy, is associated with a substantial reduction in risk of breast cancer (32–34). Furthermore, in young patients with early breast cancer, chemotoxic damage to the ovaries associated with systemic chemotherapy carries a high risk of amenorrhea and early menopause which is believed to provide benefit in terms of cancer outcomes; this benefit comes, however, at the cost of reduced fertility. More recently, a meta-analysis by the EBCTCG demonstrated definitively that ovarian ablation as a single intervention reduces risk of recurrence for women aged less than 50 years with axillary node-positive and node-negative disease (15-year survival was 52.4% for those undergoing ovarian ablation *versus* 46.1% in those who did not) (35). Thus, there is a clear link between the reduction of ovarian function, with corresponding reduction in circulating estrogens, and improved outcomes in breast cancer.

In the modern clinical setting, as well as complete surgical ovarian ablation, OFS can be achieved through the administration of luteinizing hormone (LH)-releasing hormone (LHRH) agonists (LHRHa; also known as gonadotropin-releasing hormone [GnRH] agonists, GnRHa) (36) or *via* radiation therapy. LHRH [also known as GnRH and gonadorelin (37)] is released from the hypothalamus and acts on G protein-coupled receptors (GnRH receptor type 1, GnRHR1) in the pituitary to increase the production of follicle-stimulating hormone (FSH) and LH, which, in turn, stimulates the release of E2 by the ovaries (38). LHRHa act by mimicking the effects of LHRH at the GnRHR1 (**Figure 1A**). Owing to their specific affinity for LHRH receptors, when first administered LHRHa initially produce a surge in ovarian hormones that can be accompanied by adverse effects, such as hot flashes. However, long-term administration of LHRHa reduces ovarian hormone production and secretion by causing a downregulation and desensitization of LHRH receptors in pituitary gonadotropic cells (39). The resulting reduction of circulating estrogens slows the growth of HR+ tumors.

Initially, development of clinically useful LHRHa was complicated by their short half-life, but by modification of several amino acids found in the human LHRH peptide, long-acting agonists have been successfully developed and have become useful agents in the treatment of both prostate and breast cancer. The most used LHRHa are the GnRHR1 agonists goserelin (Zoladex^®^) (40), triptorelin (Decapeptyl^®^) (41), and leuprorelin (Lupron^®^) (**Table 1**).

**Table 1:** Luteinizing hormone releasing-hormone agonists.

Generic name	Brand name	IUPAC condensed sequence	Dosing frequency	Formulations^a^	Needle characteristics
Gonadorelin (hGnRH)		H-Pyr-His-Trp-Ser-Tyr-Gly-Leu-Arg-Pro-Gly-NH2			
Goserelin acetate	Zoladex^®^	H-Pyr-His-Trp-Ser-Tyr-D-Ser(tBu)-Leu-Arg-Pro-NHNHCONH2	1- or 3- monthly	SC slow-release solid implants containing goserelin acetate equivalent to 3.6 mg and 10.8 mg goserelin, respectively	14 or 16 gauge
Triptorelin acetate	Decapeptyl SR^®^	H-Pyr-His-Trp-Ser-Tyr-D-Trp-Leu-Arg-Pro-Gly-NH2	1-, 3-, or 6-monthly	Powder and solvent to be reconstituted for IM injection containing triptorelin pamoate equivalent to 3.75 mg, 11.25 mg, and 22.5 mg triptorelin, respectively	19–21 gauge
Leuprorelin acetate	Eligard^®^ Lupron^®^ Lupron Depot^®^ Prostap 3^®^ Leuprorelin Sandoz^®^	H-Pyr-His-Trp-Ser-Tyr-D-Leu-Leu-Arg-Pro-NHEt	1-, 3-, 4-, or 6-monthly	Powder and solvent to be reconstituted for SC or IM depot injection containing leuprorelin acetate equivalent to 7.5 mg, 22.5 mg, and 45 mg leuprorelin, respectively SC slow-release solid implants containing leuprorelin acetate equivalent to 3.6 mg and 5 mg leuprorelin, respectively	14 gauge (implant) 21–23 gauge (SC) 21 gauge (IM)

a Not all formulations listed are licenced for use in both prostate and breast cancer. Not all available formulations are listed.

hGnRH, human gonadotropin-releasing hormone; IM, intramuscular; IUPAC, International Union of Pure and Applied Chemistry; SC, subcutaneous.

### Efficacy of OFS Combined With Adjuvant Endocrine Therapy

In a 2005 EBCTCG review examining 10- and 15-year disease recurrence rates and mortality in 7,601 women aged less than 50 years, benefits of OFS (via ovarian ablation or suppression with LHRHa) were observed only when OFS was given in the absence of other systemic treatments (42) and OFS did not add further benefit to that of adjuvant tamoxifen alone. However, studies included in this review may have been confounded by clinical selection criteria; some trials covered by this analysis included patients with HR− tumors and women receiving adjuvant chemotherapy, which can, on its own, produce OFS capable of masking the effects of specific OFS treatments. In contrast, a meta-analysis by the LHRH-agonists in Early Breast Cancer Overview group in 2007 found that LHRHa given alone did not significantly decrease disease recurrence (28.4% relative reduction; 95% confidence interval [CI] −50.5%, 3.5%) or death after recurrence (17.8%; 95% CI −52.8%, 42.9%) but LHRHa given in combination with tamoxifen, chemotherapy or both reduced disease recurrence and death after recurrence *versus* those therapies alone (36). In contrast to that finding, an Adjuvant Breast Cancer Trials Collaborative Group (ABCTCG) trial found no significant benefit of the addition of OFS to 5-years of tamoxifen treatment in premenopausal patients with early breast cancer (43) and the Zoladex in Pre-menopausal Patients (ZIPP) trial showed no significant difference between 2 years of treatment with tamoxifen plus goserelin *versus* 2 years of tamoxifen alone (44).

Owing to these and other contrasting findings (45), the utility of OFS as an adjuvant therapy in combination with other endocrine agents in premenopausal patients has long been contested. In recent years, several trials have sought to provide clarity over the question of whether the addition of OFS to tamoxifen or AIs provides real added benefit in the adjuvant setting for premenopausal patients with HR+ breast cancer.

#### Tamoxifen Plus OFS *Versus* Tamoxifen Alone

The phase 3 Eastern Cooperative Oncology Group (ECOG) 3193 trial (E-3193; INT-0142) (**Table 2**) comparing standard 5-year tamoxifen treatment with 5 years of tamoxifen plus OFS (surgical ablation, radiation, goserelin, or leuprolide acetate) in premenopausal women with node-negative, HR+ breast cancer found no significant difference between tamoxifen alone and tamoxifen plus OFS in the primary endpoints of disease-free survival (DFS; 5-year rate: 87.9% *versus* 89.7%) and OS (95.2% *versus* 97.6%) (46). However, this trial may have been confounded by its size, the relatively low-risk population it included, and the unknown HER2 status of most enrolled patients.

**Table 2:** Overview of trials evaluating the addition of OFS to adjuvant endocrine therapy in premenopausal women with HR+ breast cancer.

Trial name	Randomized patients, N	Clinical characteristics	Follow-up, years	Age	Treatment arms	Outcomes
E-3193 (46) (INT-0142) Phase 3	345	Premenopausal Node-negative HR+ BC	9.9	Median age, 45 years	Tamoxifen *vs.* tamoxifen plus OFS (radiation therapy, surgical ablation, or goserelin 3.6 mg or leuprolide 3.75 mg acetate, 4-weekly) Adjuvant treatment duration 5 years	5-year DFS tamoxifen, 87.9% 5-year DFS tamoxifen plus OFS, 89.7% DFS HR 1.17 (95% CI 0.64–2.12) 5-year OS tamoxifen, 95.2% 5-year OS tamoxifen plus OFS, 97.6% OS HR 1.19 (95% CI 0.52–2.70)
ASTRRA (47)	1,282	HR+ BC Retained or regained premenopausal status for 24 months after ending neoadjuvant or adjuvant chemotherapy	5	Median age, 40 years	Tamoxifen alone *vs.* tamoxifen plus OFS (3.6 mg goserelin, 4-weekly) Adjuvant tamoxifen for 5 years plus 2 years OFS	5-year DFS tamoxifen, 87.5% 5-year DFS tamoxifen plus OFS, 91.1% DFS HR 0.69 (95% CI 0.48–0.97); P = 0.033 5-year OS tamoxifen, 97.8% 5-year OS tamoxifen plus OFS, 99.4% OS HR 0.31 (95% CI 0.10–0.94); P = 0.029
SOFT (48) Phase 3	3,066	Premenopausal HR+ early BC	5.6	Median age, 43 years	Tamoxifen *vs.* tamoxifen plus OFS (bilateral oophorectomy, ovarian radiation, or triptorelin 3.75 mg, 4-weekly) *vs.* exemestane plus OFS Adjuvant treatment duration 5 years	5-year DFS tamoxifen, 84.7% 5-year DFS tamoxifen plus OFS, 86.6% DFS HR 0.83 (95% CI 0.66–1.04); P = 0.10 5-year OS tamoxifen, 95.1% 5-year OS tamoxifen plus OFS, 96.7% OS HR 0.74 (95% CI 0.51–1.09); P = 0.13
SOFT (49) Phase 3	3,066	Premenopausal HR+ early BC	8	Median age, 43 years	Tamoxifen *vs.* tamoxifen plus OFS (bilateral oophorectomy, ovarian radiation, or triptorelin 3.75 mg, 4-weekly) *vs.* exemestane plus OFS Adjuvant treatment duration 5 years	8-year DFS tamoxifen, 78.9% 8-year DFS tamoxifen plus OFS, 83.2% DFS HR 0.76 (95% CI 0.62–0.93); P = 0.009 8-year OS tamoxifen, 91.5% 8-year OS tamoxifen plus OFS, 93.3% OS HR 0.67 (95% CI 0.48–0.92); P = 0.01 8-year DFS exemestane plus OFS, 85.9% DFS HR *vs.* tamoxifen alone 0.65 (95% CI 0.53–0.81) 8-year OS exemestane plus OFS, 92.1% OS HR *vs.* tamoxifen alone 0.85 (95% CI 0.62–1.15)
E-5188 (50) (INT-101) Phase 3	1,503	Premenopausal Node-positive HR+ BC	9.6	<40 years, 438 (29%) ≥40 years, 1,065 (71%)	CAF chemotherapy alone *vs.* CAF chemotherapy followed by OFS (goserelin 3.6 mg, 4-weekly) *vs.* CAF followed by OFS plus tamoxifen Adjuvant treatment duration 5 years	9-year DFS CAF alone, 57% 9-year DFS CAF plus goserelin, 60% 9-year DFS CAF plus goserelin and tamoxifen, 68% DFS HR CAF plus goserelin *vs.* CAF plus goserelin plus tamoxifen 0.74 (95% CI 0.60–0.91); P < 0.01 DFS HR CAF *vs.* CAF plus goserelin 0.93 (95% CI 0.76–1.12); P = 0.22 9-year OS CAF alone, 70% 9-year OS CAF plus goserelin, 73% 9-year OS CAF plus goserelin and tamoxifen, 76% OS HR CAF plus goserelin *vs.* CAF plus goserelin plus tamoxifen 0.91 (95% CI 0.71–1.15); P = 0.21 OS HR CAF *vs.* CAF plus goserelin 0.88 (95% CI 0.70–1.11); P = 0.14
ABCSG-12 (51) Phase 3	1,803	Premenopausal Stage 1–2 HR+ BC and low risk of disease recurrence	7.9	Median age, 45 years	Tamoxifen plus OFS (goserelin 3.6 mg, 4-weekly) plus zoledronic acid *vs.* anastrazole plus OFS plus zoledronic acid Adjuvant treatment duration 3 years	7.9-year DFS tamoxifen plus goserelin, 117 events 7.9-year DFS anastrozole plus goserelin, 134 events DFS HR, 1.13 (95% CI 0.88–1.45); P = 0.335 7.9-year OS tamoxifen plus goserelin, 33 events 7.9-year OS anastrozole plus goserelin, 53 events OS HR, 1.63 (95% CI 1.05–2.52); P = 0.030
STAGE (52) Phase 3	204	Premenopausal HR+ early BC	0.5		Tamoxifen plus OFS (goserelin 3.6 mg, 4-weekly) *vs.* anastrozole plus OFS Neoadjuvant treatment duration 24 weeks	Overall (complete or partial) tumor response rate tamoxifen plus goserelin, 50.5% Overall tumor response rate anastrozole plus goserelin, 70.4% Difference between groups, 19.9% (95% CI 6.5–33.3%); P = 0.004
TEXT (49) Phase 3	2,672	Premenopausal HR+ early BC	8	Median age, 44 years	Tamoxifen plus OFS (bilateral oophorectomy, ovarian radiation or triptorelin 3.75 mg, 4-weekly) *vs.* exemestane plus OFS Adjuvant treatment duration 5 years	See combined SOFT + TEXT analysis
SOFT + TEXT (53) Phase 3	4,690^b^	Premenopausal HR+ early BC	5.7	Median age, 43 years	Tamoxifen *vs.* tamoxifen plus OFS (bilateral oophorectomy, ovarian radiation, or triptorelin 3.75 mg, 4-weekly) *vs.* exemestane plus OFS Adjuvant treatment duration 5 years	5-year DFS tamoxifen plus OFS, 87.3% 5-year DFS exemestane plus OFS, 91.1% DFS HR 0.72 (95% CI 0.60–0.85); P < 0.001 5-year OS tamoxifen plus OFS, 96.9% 5-year OS exemestane plus OFS, 95.9% OS HR 1.14 (95% CI 0.86–1.15); P = 0.37
SOFT + TEXT^a^ (49), Phase 3	4,690^b^	Premenopausal HR+ early BC	8	Median age, 43 years	Tamoxifen *vs.* tamoxifen plus OFS (bilateral oophorectomy, ovarian radiation, or triptorelin 3.75 mg, 4-weekly) *vs.* exemestane plus OFS Adjuvant treatment duration 5 years	8-year DFS tamoxifen plus OFS, 82.8% 8-year DFS exemestane plus OFS, 86.8% DFS HR 0.77 (95% CI 0.67–0.90); P < 0.001 8-year OS tamoxifen plus OFS, 93.3% 8-year OS exemestane plus OFS, 93.4% OS HR 0.98 (95% CI 0.79–1.22); P = 0.84
HOBOE (54) Phase 3	710^c^	Premenopausal HR+ BC	5.3	Median age, 45 years	Tamoxifen plus OFS (triptorelin 3.75 mg, 4-weekly) *vs.* letrozole plus OFS Adjuvant treatment duration 5 years	5-year DFS tamoxifen plus OFS, 85.4% 5-year DFS letrozole plus OFS, 93.2% DFS HR 0.72 (95% CI 0.48–1.07); P = 0.06 5-year death rate tamoxifen plus OFS, 4.8% 5-year death rate letrozole plus OFS, 3.1% OS HR not reported; P = 0.14

a Includes patients (N = 1014) from the exemestane plus OFS arm of SOFT.

b Number of patients included in combined analysis after exclusions.

c The total number of patients randomized in the trial was 1,065. The letrozole plus OFS plus zoledronic acid group (N = 355) is not included.

BC, breast cancer; CAF, cyclophosphamide, adriamycin, fluorouracil; CI, confidence interval; DFS, disease-free survival; HR, hazard ratio; HR+, hormone receptor positive; OFS, ovarian function suppression; OS, overall survival.

The ASTRRA trial (**Table 2**) also evaluated the efficacy of adding OFS (goserelin) to 5 years of adjuvant tamoxifen, this time in patients with HR+ breast cancer who retained or regained premenopausal status following neoadjuvant/adjuvant chemotherapy (55). In these patients, who had a higher risk of disease recurrence and previous chemotherapy, the addition of OFS to tamoxifen resulted in a significant improvement in 5-year DFS (91.1% *versus* 87.5% with tamoxifen alone). A significant improvement in OS was also observed in the OFS plus tamoxifen group (99.4% *versus* 97.8%) (47), although this finding is confounded by the small number of events (four in the tamoxifen plus OFS group and 14 in the tamoxifen only group).

Conflicting results were observed in the initial analysis of the Suppression of Ovarian Function Trial (SOFT) (**Table 2**), conducted by the International Breast Cancer Study Group (IBCSG). In SOFT, premenopausal patients with HR+ early breast cancer were randomized to receive exemestane plus OFS (bilateral oophorectomy, ovarian radiation, or triptorelin), tamoxifen plus OFS, or tamoxifen alone (48, 49, 56). In the primary analysis, performed at 5.6 years follow-up, no significant difference was observed between patients who received tamoxifen plus OFS and those who received tamoxifen alone for DFS (5-year event rate 86.6% *versus* 84.7%) or OS (5-year event rate 96.7% *versus* 95.1%) (48). Thus, at primary analysis, no benefit of adding OFS to tamoxifen was observed in the overall patient group, which included premenopausal women of all ages and all prior chemotherapy statuses. However, in SOFT, 90% of the deaths occurred in patients who had received prior chemotherapy, which may have confounded the overall results. Indeed, in patients who had not received prior chemotherapy, 5-year OS rates exceeded 99% in both treatment groups (48), whereas in patients who had received prior chemotherapy, tamoxifen plus OFS led to a significant improvement in OS versus tamoxifen alone (94.5% *versus* 90.9%, hazard ratio [HR]: 0.64, 95% CI 0.42–0.96). Thus, the initial analysis of SOFT demonstrated some benefit of the addition of OFS to tamoxifen, but only in terms of OS for patients who had received prior chemotherapy.

An updated analysis of SOFT, with 8-years of follow-up, subsequently showed a significant improvement in both DFS (8-year rate 83.2% *versus* 78.9%) and OS (8-year rate 93.3% *versus* 91.5%) for all patients who received tamoxifen plus OFS *versus* tamoxifen alone (49). While the relative benefits of tamoxifen plus OFS were similar regardless of prior chemotherapy, the absolute benefits were greater in those patients who remained premenopausal having received prior chemotherapy (49). Clinico-pathological features in these patients, including younger age, may have contributed to a higher risk of disease recurrence. Indeed, DFS in this cohort was 5.3% higher in patients who received tamoxifen plus OFS than in patients who received tamoxifen alone.

The most recent data from SOFT therefore support the addition of OFS to tamoxifen in the adjuvant setting for higher-risk women who remain premenopausal after receiving adjuvant chemotherapy. A recent Cochrane Library systematic review and meta-analysis conducted by Bui and colleagues of 11 studies including 10,374 women supports this conclusion, having demonstrated that addition of OFS to tamoxifen resulted in a significant reduction in mortality (HR: 0.86, 95% CI 0.78–0.94) (57).

#### Tamoxifen Plus OFS *Versus* OFS Alone

The ECOG 5188 trial (E5188, INT-101) (**Table 2**) compared 5 years of adjuvant tamoxifen plus OFS (goserelin), OFS alone, or no adjuvant endocrine therapy in premenopausal women with node-positive, HR+ breast cancer who had previously received cyclophosphamide, doxorubicin, fluorouracil (CAF) chemotherapy (50). The addition of tamoxifen to OFS significantly improved 9-year DFS (68% *versus* 60%; P < 0.01) but not 9-year OS (76% *versus* 73%; P = 0.21) compared with OFS in the overall population. Results of a retrospective subgroup analysis also showed that combining tamoxifen and OFS seemed to provide superior DFS outcomes *versus* OFS alone both in women aged less than 40 years (64% *versus* 55%) and those aged 40 years and older (69% *versus* 62%) (50).

#### Anastrozole Plus OFS *Versus* Tamoxifen Plus OFS

The Austrian Breast and Colorectal Cancer Study Group (ABCSG)-12 (**Table 2**) trial compared 3 years of treatment with either the AI anastrozole plus OFS (goserelin) or tamoxifen plus OFS in premenopausal women with stage 1–2 HR+ breast cancer and a low risk of disease recurrence (51, 58, 59). Although there was no significant difference in DFS between treatment groups, a higher risk of death was observed for patients who received anastrozole than for those who received tamoxifen (53 *versus* 33 events; HR: 1.63, 95% CI 1.05–2.52; P = 0.03). Therefore, although this study did not compare the benefits of either tamoxifen or AI plus OFS versus tamoxifen alone, the data suggest that combining OFS with tamoxifen provides greater benefit than combining it with AIs.

In contrast, the phase 3 STAGE study comparing anastrozole plus OFS (goserelin) with tamoxifen plus OFS, given in the neoadjuvant setting to a premenopausal HR+/HER2− Japanese patient cohort, found a significantly higher tumor response rate for anastrozole plus OFS *versus* tamoxifen plus OFS (70.4% *versus* 50.5%). However, this study was relatively small (N = 204 patients) and, compared with a typical adjuvant treatment duration of 5 years, the neoadjuvant treatment period was short (24 weeks) (52).

#### Exemestane Plus OFS *Versus* Either Tamoxifen Plus OFS or Tamoxifen Alone

Further conflicting evidence regarding whether OFS is more effective when combined with an AI *versus* with tamoxifen comes from two studies that compared exemestane plus OFS with tamoxifen plus OFS – the SOFT trial and the contemporaneous phase 3 Triptorelin and Exemestane Trial (TEXT) (56). A combined analysis of tamoxifen from SOFT and TEXT after a median follow-up of 5.7 years found that DFS was significantly higher for exemestane plus OFS than for tamoxifen plus OFS (5-year DFS 91.1% *versus* 87.3%), but there was no significant difference in OS (**Table 2**) (53). When the duration of follow-up was increased to a median of 8 years, a similar pattern of results was obtained (**Table 2**) (49, 53).

In SOFT, comparisons were also made between the exemestane plus OFS arm and the tamoxifen alone arm. These analyses showed that the 8-year DFS rate was significantly higher with combination therapy than with tamoxifen monotherapy (85.9% and 78.9%, respectively) whereas the OS was similar for both groups (**Table 2**) (49).

Similar to what was observed in the tamoxifen plus OFS *versus* tamoxifen alone arms of SOFT described earlier, the addition of OFS to exemestane provided greater absolute benefits in DFS in patients who had received prior chemotherapy. In this cohort, DFS was 9% higher in patients who had received exemestane plus OFS than in patients who had received tamoxifen alone (49).

The results of SOFT and SOFT/TEXT, after 9 years of follow-up, suggest that the addition of OFS to tamoxifen results in significantly higher rates of DFS and OS than tamoxifen alone, and that addition of OFS to an AI leads to significantly higher rates of DFS than tamoxifen alone, particularly in patients at high risk of disease recurrence who had received prior chemotherapy (48, 49, 60). Addition of OFS to an AI also produced a greater absolute benefit in DFS than did addition of OFS to tamoxifen, but no greater absolute benefit in OS. Therefore, considering the lack of superiority of an AI (plus OFS) over tamoxifen (plus OFS) on OS, the decision to choose an AI plus OFS must be weighed against additional complications of using AIs in premenopausal women (see below).

#### Letrozole Plus OFS *Versus* Tamoxifen Plus OFS

Further evidence for a lack of difference between AIs over tamoxifen when combined with OFS comes from the phase 3 HOrmonal BOne Effects (HOBOE) trial (61) (**Table 2**). In two arms of the three-arm trial, premenopausal women with HR+ breast cancer were randomized to receive adjuvant letrozole plus OFS (triptorelin) or tamoxifen plus OFS. A numerically greater benefit in 5-year DFS rates was observed for letrozole plus OFS *versus* tamoxifen plus OFS although the difference did not reach statistical significance (54) (**Table 2**). There was no significant difference in 5-year OS between the two groups.

#### Conclusion

**Figure 2:**
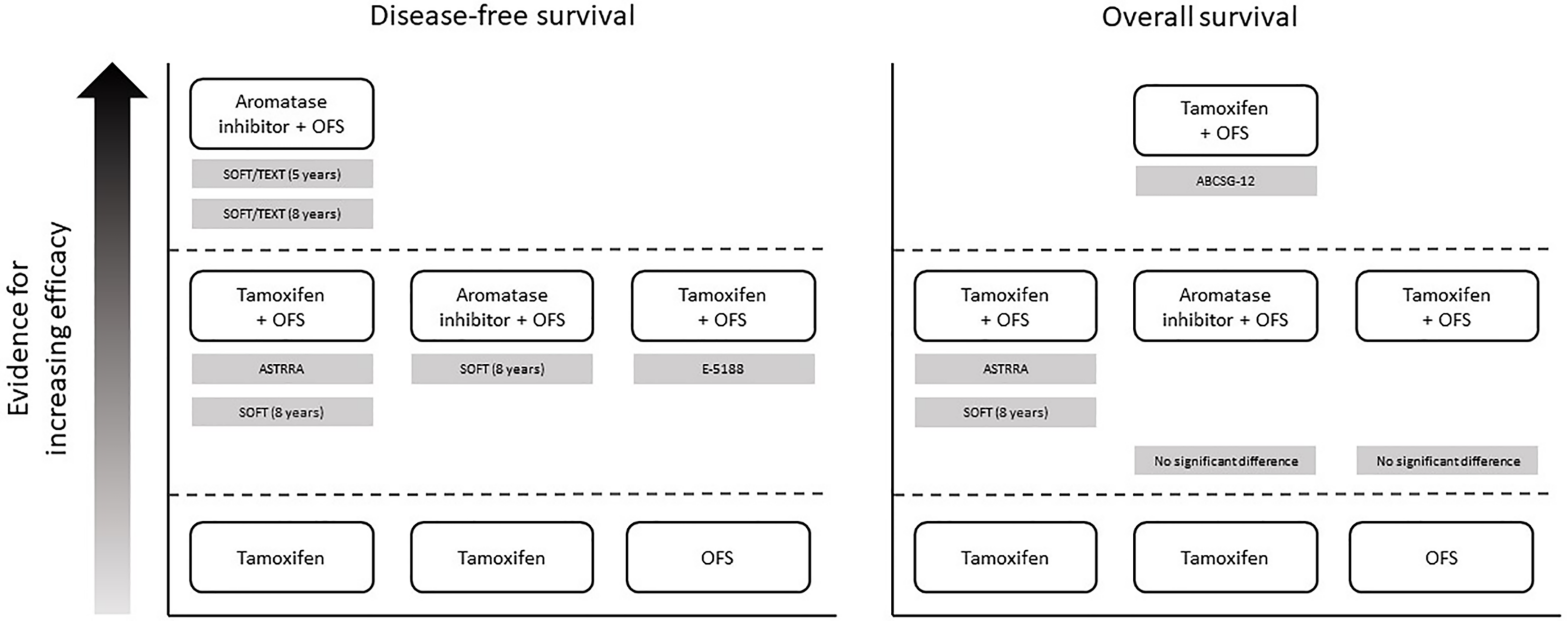
Overview of evidence supporting the addition of OFS to endocrine therapy in premenopausal women with HR+ breast cancer. In each panel, treatments that are positioned higher up in the figure have been shown to be more effective than treatments positioned lower down (directly below). Supporting studies are detailed in gray boxes. Note that the figure does not present data from studies demonstrating equivalent efficacy between treatments – see main text and **Table 2** for full results of all studies. ABCSG, Austrian Breast and Colorectal Cancer Study Group; HR+, hormone receptor-positive; OFS, ovarian function suppression; SOFT, Suppression of Ovarian Function Trial; TEXT, Triptorelin and Exemestane Trial.

While ECOG 3193 found no benefit in adding OFS to endocrine therapy, other studies have shown that this combination can improve survival outcomes, *versus* either OFS alone (ECOG 5188) or endocrine therapy alone (SOFT, SOFT/TEXT, and ASTRRA). There is also some evidence to suggest that combining OFS with an AI may lead to more favorable outcomes than combining OFS with tamoxifen (STAGE and SOFT/TEXT; **Figure 2**). However, results from other studies indicate that the opposite may be true (ABCSG OS data) or that there is no difference between the two endocrine therapies (ABCSG DFS data and HOBOE). Overall, the most recent available evidence suggests that OFS added to either tamoxifen or AIs can provide significant benefit in premenopausal patients with less favorable clinicopathological characteristics, such as those who have received previous chemotherapy. Since ER– tumors are not sensitive to ovarian E2 secretion, American Society for Clinical Oncology guidelines state that there is no role for OFS as adjuvant therapy in ER– breast cancers (62).

### Efficacy of OFS Combined With Adjuvant Chemotherapy for Fertility Preservation in Premenopausal Women With Breast Cancer

Premenopausal women diagnosed with early breast cancer associated with unfavorable clinico-pathological features are candidates for treatment with systemic chemotherapy. In this group of patients, adjuvant chemotherapy has demonstrated clinical effectiveness in reducing risk of breast cancer relapse and death (16). However, in these, typically younger, women there is a risk of long-lasting and impactful toxicities associated with chemotherapy. One such risk is of cytotoxic damage to the ovaries and therapy-induced amenorrhea which can be permanent and can cause infertility. Estimates of the rate of chemotherapy-induced amenorrhea, for regimens including cyclophosphamide, vary between 20% and 70% in premenopausal women aged under 40 years but can rise to near 100% in older premenopausal patients (63). This is of increasing concern because, in many countries, the age of childbearing is increasing; a change that is accompanied by an increased risk of developing breast cancer. The potential for loss of fertility due to treatment can have serious psychological effects on women and can, therefore, influence treatment decisions taken at diagnosis.

Temporary OFS, using LHRHa, during adjuvant chemotherapy has been developed as a potential option to prevent chemotherapy-mediated gonadotoxicity and premature ovarian failure (POF) to maintain fertility in women of childbearing age undergoing breast cancer treatment. This approach is also recommended by the European Society for Medical Oncology in female patients who wish to preserve ovarian function and/or fertility while undergoing cancer treatment (64). In the past 15 years, several randomized clinical trials have attempted to answer the question of whether LHRHa administration during chemotherapy is effective in preventing POF and preserving fertility. The three largest phase 3 studies to date (> 200 patients each) are the Prevention of Early Menopause Study (POEMS) (65, 66), the Prevention of Menopause Induced by Chemotherapy: A Study in Early Breast Cancer Patients-Gruppo Italiano Mammella 6 (PROMISE-GIM6) trial (67, 68), and the Anglo Celtic OPTION trial (69). In contrast with the use of OFS as an adjuvant therapy, in the POEMs and OPTION trials, patients with ER– breast cancer were enrolled. This strategy was adopted in these trials owing to concerns about the ability of concurrent endocrine therapy to reduce the efficacy of chemotherapy. However, recent results in the TEXT trial suggest LHRHa are likely to be suitable for use concurrently with chemotherapy in women for HR+ breast cancer.

In the POEMS trial (**Table 3**), patients received either adjuvant/neoadjuvant chemotherapy alone or chemotherapy plus OFS (goserelin). After 2 years of follow-up, the POF rate was significantly lower in the group that received OFS (8%) *versus* the group that received only chemotherapy (22%) (65). At 5 years of follow-up, the rate of pregnancies was also significantly higher in the OFS-treated group (5-year cumulative incidence: 23.1% *versus* 12.2%) (66).

**Table 3:** Overview of largest trials evaluating the addition of OFS to adjuvant chemotherapy for fertility preservation in premenopausal women with breast cancer.

Trial name	Randomized patients, N	Clinical characteristics	Follow up, years	Median age	Treatment arms	Outcomes
POEMS/SWOG (65, 66) Phase 3	218	Premenopausal Early-stage BC	2 (POF) 5 (pregnancy)	Overall, 38 years Chemotherapy alone, 38.7 years Chemotherapy plus OFS, 37.6 years	Neoadjuvant/adjuvant cyclophosphamide-containing chemotherapy alone *vs.* chemotherapy plus 3.6 mg 4-weekly goserelin	POF defined as amenorrhea for the preceding 6 months and FSH levels in the postmenopausal range at 2 years^a^ POF rate chemotherapy alone, 22% POF rate chemotherapy plus OFS, 8% POF OR: 0.30 (95% CI 0.09–0.97); P = 0.04 5-year cumulative incidence of pregnancy: chemotherapy alone, 12.2% chemotherapy plus OFS, 23.1% OR: 2.34 (95% CI 1.07–5.11); P = 0.03
PROMISE-GIM6 (67, 68) Phase 3	281	Premenopausal BC	1 (POF) 7.3 (menstrual resumption; pregnancy)	39 years	Chemotherapy alone *vs.* chemotherapy plus 3.75 mg 4-weekly triptorelin	POF defined as no resumption of menstrual activity or the presence of postmenopausal levels of FSH and E2 for 1 year after the end of chemotherapy POF rate chemotherapy alone, 25.9% POF rate chemotherapy plus OFS, 8.9% Absolute difference: −17%; P < 0.001 5-year cumulative incidence of menstrual resumption: chemotherapy alone, 64.0% chemotherapy plus OFS, 72.6% 5-year incidence of pregnancy: chemotherapy alone, 1.6% chemotherapy plus OFS, 2.1%
Anglo Celtic Group OPTION (69)	227	Early-stage BC	1–2	Chemotherapy alone, 38.8 years Chemotherapy plus OFS, 37.9 years	Cyclophosphamide- and/or anthracycline-containing chemotherapy alone *vs.* chemotherapy plus 3.6 mg 4-weekly goserelin	Primary outcome was amenorrhea at 12–24 months after end of chemotherapy Amenorrhea rate chemotherapy alone, 38% Amenorrhea rate chemotherapy plus OFS, 22% (P = 0.015) POF defined as the presence of amenorrhea and elevated FSH (> 25 IU/L) POF rate chemotherapy alone, 34.8% POF rate chemotherapy plus OFS, 18.5%

a POF was evaluated in 135 patients for whom data were available at 2 years.

BC, breast cancer; CI, confidence interval; E2, estradiol; FSH, follicle-stimulating hormone; IU, international units; OFS, ovarian function suppression; OR, odds ratio; POF, premature ovarian failure.

The PROMISE-GIM6 trial (**Table 3**) was an Italian study that randomized premenopausal breast cancer patients to receive either chemotherapy alone or chemotherapy plus OFS (triptorelin, starting 1 week prior to chemotherapy). The rate of POF 12 months after the end of chemotherapy was significantly higher in the chemotherapy alone group than in the chemotherapy plus OFS group (25.9% *versus* 8.9%) (67). After a median follow-up of 7.3 years, the benefit of OFS was retained, with a 5-year cumulative incidence of menstrual resumption of 64.0% *versus* 72.6%, and a 5-year incidence of pregnancy of 1.6% *versus* 2.1% (68).

Further reassuring evidence regarding the safety of this strategy come from the recently published final analysis of the study (median follow-up of 12.4 years), in which the 10-year cumulative pregnancy incidence was 3.2% in patients receiving chemotherapy alone, compared with 6.5% in patients receiving chemotherapy plus OFS. Importantly, 80% of the trial population had HR+ disease, yet no interaction between treatment effect and HR status was observed (70).

In the Anglo Celtic Group OPTION trial (**Table 3**), patients with early-stage breast cancer were randomized to receive either chemotherapy alone or chemotherapy plus OFS (goserelin). In the primary analysis, the prevalence of amenorrhea was significantly reduced with the addition of OFS, from 38% in the chemotherapy alone group to 22% in the chemotherapy plus OFS group (P = 0.015) (69). POF was also higher in the chemotherapy alone group (34.8% *versus* 18.5%), while the number of pregnancies was lower (six *versus* nine) (69).

Thus, the three largest trials to date support addition of LHRHa to chemotherapy to reduce POF and to help in maintaining fertility in premenopausal women. Importantly, the addition of LHRHa to achieve preservation of ovarian function does not have detrimental effects on the effectiveness of the chemotherapy. For example, in the PROMISE-GIM6 trial, the estimated 5-year DFS rates were 80.5% (95% CI 73.1–86.1) for chemotherapy plus triptorelin *versus* 83.7% (95% CI 76.1–89.1) for chemotherapy alone (HR: 1.17, 95% CI 0.72–1.92, P = 0.519) (71). Several smaller trials, including the GBG73 ZORO trial (72), a study by Badawy and colleagues (73), and several meta-analyses that support this conclusion were recently summarized in detail in a comprehensive review by Lambertini and colleagues (74).

### Practicalities of Using LHRHa in Treatment of Breast Cancer

When determining the most appropriate adjuvant endocrine therapy for individual patients, the potential benefits of the addition of LHRHa must be weighed against increased rates of side effects and practical aspects of using these drugs. The effects of addition of OFS to tamoxifen or AIs on adverse events and patient-reported outcomes have been reported in the E-3193, SOFT, ZIPP, and OPTION trials (46, 75–78). Overall, evidence from these trials suggests that, in premenopausal patients, addition of LHRHa to tamoxifen is associated with worse endocrine symptoms and sexual function, which is particularly problematic in the population of younger women who gain most benefit from these drugs. Analysis of the combined SOFT and TEXT showed that when OFS was added to tamoxifen or exemestane, reported grade 3 or 4 adverse events increased (tamoxifen 24.6% *versus* tamoxifen plus OFS 31.0% *versus* exemestane plus OFS 32.3%) and patients reported considerable worsening from baseline in key endocrine symptoms (including hot flushes, depression, sweating, fatigue, and insomnia). No overall difference in quality of life was found between tamoxifen and AI (49, 79). In the combined analysis of SOFT and TEXT, 19% of patients overall stopped treatment with LHRHa earlier than the 5-year planned treatment duration (tamoxifen plus LHRHa 19.6%, exemestane plus LHRHa 18.3%) (49); this increased to 23% at 4-years in the most high-risk patient group aged under 35 years (80). In the patients receiving tamoxifen plus LHRHa, 8.1% had an adverse event related to a reaction at the drug injection site; this was 7.5% in those receiving exemestane plus LHRHa compared with 0.4% in those receiving only tamoxifen (49). Rates of early discontinuation of oral endocrine therapy in SOFT and TEXT were 21.5% overall, with discontinuation higher in those assigned exemestane plus OFS (23.7%) than in those receiving tamoxifen plus OFS (19.3%) (49). However, recently reported results from the OPTION trial suggest that when goserelin is added to chemotherapy to provide ovarian function protection in premenopausal women with early breast cancer the detrimental effects experienced on quality of life are short-lived. Within 24 months, the majority of patient-reported outcomes in individuals receiving goserelin with chemotherapy did not differ from those receiving chemotherapy alone (78).

Another potential problem when adding LHRHa to AIs is the risk associated with incomplete estrogen suppression. AIs are more effective than tamoxifen in postmenopausal women and, when combined with OFS in premenopausal women, have been associated with greater improvements in DFS and OS *versus* tamoxifen plus OFS for some patients. However, in premenopausal women, most estrogen production occurs in the ovaries. For AIs to be fully effective, unlike with tamoxifen, complete suppression of ovarian function is required. Numerous studies have demonstrated the ability of LHRHa to significantly suppress E2 levels, but it remains unclear whether the degree of E2 suppression is sufficient to permit combination with AIs in some high-risk premenopausal women. In fact, the SOFT-Estrogen (SOFT-EST) prospective study, which measured E2 levels in 116 patients, found that between 17–25% of patients receiving exemestane plus triptorelin had E2 levels above the threshold target level of ≤ 2.72 pg/mL during the 12-month study (81). Thus, when deciding to use an AI combined with LHRHa to treat those patients with the worst prognostic features, clinicians should be aware of the potential for incomplete OFS and closely monitor patient E2 levels during treatment. In real-world situations in which E2 monitoring is not available, tamoxifen plus LHRHa should be considered for patients at higher risk of incomplete OFS, including younger women, those who have not received prior chemotherapy, and those with a high body mass index (BMI). Indeed, in the ABCSG-12 trial, secondary analysis showed that patients with a BMI ≥ 25 kg/m^2^ receiving anastrozole plus goserelin had a 50% increased risk of disease recurrence (HR: 1.49, 95% CI 0.93–2.38) and 3-fold increase in risk of death (HR: 3.03, 95% CI 1.35–6.82) compared with those receiving tamoxifen plus goserelin (82). This may be due to incomplete OFS in patients with higher BMI but could also be the result of increased ER activation by other factors, such as insulin/insulin-like growth factor, that are increased in overweight patients. The confounding factors in the worse prognosis for overweight patients require further exploration in future randomized control trials.

One approach to overcoming some of the problems associated with the use of LHRHa in the clinic has been the introduction of long-acting drug formulations (83). In prostate cancer, in which LHRHa including goserelin, triptorelin, and leuprorelin are used to reduce circulating androgens (**Figure 1B**), long-acting drug formulations have been shown to be clinically effective and well tolerated and have been used extensively for several years (84, 85). Several long-acting formulations have been approved for use in prostate cancer, allowing dosing at 1-, 2-, 3-, 4-, 6-, and 12-month intervals (**Table 1**). Goserelin acetate (Zoladex^®^) is available as a slow-release solid implant injected subcutaneously on a monthly or three-monthly basis. Leuprorelin acetate is available in several different formulations that allow for 1-, 3-, 4-, or 6-monthly administration, including a slow-release solid implant (83). Triptorelin acetate (Decapeptyl^®^ SR) can be administered intramuscularly at 1-, 3-, or 6-monthly dosing intervals (83). Experience from use in prostate cancer has shown several advantages of solid implant formulations, including being ready to use with no need for reconstitution and the ability to be stored without refrigeration (83). Moreover, the exact dose given is known whereas for other formulations, issues can arise when reconstitution is performed incorrectly, potentially resulting in insufficient dosing (86). A final advantage of solid implants *versus* gel-like or reconstituted powder injections is the ability to remove the implant in the event of severe adverse effects of the medication. Long-acting formulations are also be preferred by patients (83).

In breast cancer, long-acting LHRHa formulations remain less commonly used than short-acting alternatives and fewer different formulations are approved for use in a smaller number of countries. For premenopausal patients with HR+ breast cancer, leuprorelin is available as a 1-, 3-, and 6-month depot formulation (Lupron^®^, Prostap 3). Early studies showed that 3-monthly administration of leuprorelin was as effective, as well tolerated, and provided similar E2 suppression as monthly administration (87). A retrospective analysis of SOFT and TEXT found that, in 201 patients randomized to receive an AI plus either 7.5 mg leuprorelin monthly or 22.5 mg leuprorelin 3-monthly, the ability to achieve ovarian ablation (defined as an E2 concentration < 40 pg/mL and an FSH concentration 23–116 mU/mL) was the same with both formulations (88). In 167 premenopausal patients with HR+ breast cancer randomized to receive either 11.25 mg leuprorelin 3-monthly or 22.5 mg leuprorelin 6-monthly, the rate of E2 suppression (to ≤ 30 pg/mL) was found to be 1.2% higher in the group using the longer-acting formulation (3-monthly formulation 96.4%; 6-monthly formulation 97.6%) without significant differences in adverse events (89). In a retrospective study by Lee and colleagues of 318 women who had previously undergone surgery for breast cancer, post-surgery treatment with 3-monthly leuprorelin acetate (11.25 mg) successfully reduced E2 levels below 30 pg/mL (mean: 4.9 pg/mL) in all patients demonstrating the effectiveness of this formulation (90).

Goserelin (Zoladex^®^) is used in premenopausal patients with breast cancer as a 3.6 mg solid implant and has more recently been approved for use as a 3-monthly 10.8 mg implant in Japan, Taiwan, Ukraine, South Korea, Indonesia, Singapore and Malaysia. Several studies have demonstrated the non-inferiority of 3-monthly *versus* monthly goserelin. In an open-label, randomized study conducted in Japan, E2 levels were measured in 170 premenopausal patients with ER+ early breast cancer who were randomized to receive either goserelin 10.8 mg given 3-monthly or goserelin 3.6 mg given monthly. After 24 weeks of treatment, serum E2 levels were 18.95 pg/mL (n = 84) for goserelin 3.6 mg and 18.32 pg/mL (n = 86) for goserelin 10.8 mg (91), demonstrating comparable OFS with both formulations; no clinically important differences in safety and tolerability were found. In a further trial conducted in India, Japan, Republic of Korea, Philippines, Thailand, and Taiwan in 222 patients with ER+ advanced breast cancer, progression-free survival (PFS) and overall response rates (ORRs) after 24 weeks were similar with goserelin 10.8 mg given 3-monthly and 3.6 mg given monthly (PFS: 10.8 mg, 61.5%; 3.6 mg, 60.2%; ORR: 10.8 mg, 23.9%; 3.6 mg, 26.9%). Similar to the previous study, E2 levels at 24 weeks were also suppressed equally by 3-monthly (10.8 mg, 20.3 pg/mL) and monthly (3.6 mg, 24.8 pg/mL) administration (92). A third study conducted in Russia and Ukraine also found non-inferiority of 3-monthly *versus* monthly goserelin, with similar PFS, ORR, and E2 suppression observed for both formulations. Finally, an ongoing phase 3 study in China, due for completion in November 2021 (NCT03658213), is investigating the non-inferiority of 3-monthly 10.8 mg goserelin in ER+/HER2− early breast cancer patients.

## Conclusions

As treatment options have rapidly expanded, management of adjuvant treatment of premenopausal women with early and advanced breast cancer has become more complicated. The most recent evidence suggests that addition of LHRHa to adjuvant endocrine therapy, with both tamoxifen and AIs, can provide significant benefits in some premenopausal patients who are at high risk of recurrence and have poor prognostic characteristics. Longer-acting depot and implant LHRHa formulations may help to overcome some of the barriers to adding OFS to endocrine therapy in the adjuvant setting in premenopausal women.

## Author Contributions

Y-SL, AW, and H-JK contributed equally to writing this review. All authors contributed to the article and approved the submitted version.

## Funding

This work was funded by AstraZeneca and medical writing support was provided by Adam Errington, PhD, Oxford PharmaGenesis, Cardiff, UK. The funder was not involved in the study design, collection, analysis, interpretation of data, the writing of this article or the decision to submit it for publication.

## Conflict of Interest

Authors received no financial compensation for this work. Y-SL has received personal fees from AstraZeneca, Eisai, EuroPharma and personal fees, research support, and other financial support from Novartis, Roche, Eli Lilly, Merck, and Pfizer. AW has received personal fees from AstraZeneca and Pfizer and research support from Otsuka Pharmaceuticals.

The remaining author declares that the research was conducted in the absence of any commercial or financial relationships that could be construed as a potential conflict of interest.

## Publisher’s Note

All claims expressed in this article are solely those of the authors and do not necessarily represent those of their affiliated organizations, or those of the publisher, the editors and the reviewers. Any product that may be evaluated in this article, or claim that may be made by its manufacturer, is not guaranteed or endorsed by the publisher.
